# Huntingtin-associated protein 1 ameliorates neurological function rehabilitation by facilitating neurite elongation through TrKA-MAPK pathway in mice spinal cord injury

**DOI:** 10.3389/fnmol.2023.1214150

**Published:** 2023-08-07

**Authors:** Li Miao, Sun Wan Qing, Lu Tao

**Affiliations:** ^1^Department of Orthopedics, Hunan Children's Hospital, Changsha, China; ^2^The School of Pediatrics, Hengyang Medical School, University of South China, Changsha, China; ^3^Key Laboratory of Pediatric Bone Science of Hunan Province, Changsha, China; ^4^Hunan Rehabilitation Hospital Third Internal Department, Changsha, China; ^5^Department of Neurosurgery, Changde Hospital, Xiangya School of Medicine, Central South University, Changde, China

**Keywords:** HAP1, spinal cord injury, neurological function rehabilitation, tropomyosin receptor kinase A (TrkA), neurite elongation

## Abstract

**Aims:**

Huntingtin-associated protein 1 (HAP1) is a neuronal protein closely associated with microtubules and might facilitate neurological function rehabilitation. This study aimed to investigate the effects of HAP1 on SCI and the underlying mechanisms.

**Methods:**

the spinal cord injury (SCI) mouse model was induced by Allen’s method. Then recombinant-HAP1 (r-HAP1) was administrated by intrathecal injection, and the BMS, Thermal nociceptive thresholds, tactile nociceptive thresholds, and neurofibrillary regeneration were identified to inspect the therapy outcome. Then NSCs were isolated from mice on embryonic day 14.5 and induced to differentiate into neurons. The efficiency of axon growth was calculated. Signaling pathway array was conducted to examine the signaling pathways in NSCs treated with r-HAP1. Antagonists and activators of TrkA were used to confirm the role of TrkA of HAP1 intervention both *in vitro* and *in vivo*.

**Results:**

r-HAP1 ameliorates the neurological function rehabilitation after SCI, and benefits the regain of Tuj in injury spinal cord. Also significantly enhances neurite growth during neuronal differentiation of NSCs; Signaling pathway array and Western blot revealed that r-HAP1 significantly activates the phosphorylation of TrkA-MAPK/ERK in NSCs. TrkA selective inhibitor GW441756 blocks r-HAP1 on TrkA-MAPK/ERK signaling pathway and detracts from axonal growth after neuronal differentiation. TrkA selective activator gambogic amide can mimic the function of r-HAP1 by activating the foregoing pathway. ERK activator U-46619 reverses the blocking effect of GW441756 on r-HAP1.

**Conclusion:**

HAP1 activates the TrkA-MAPK signaling pathway and is conducive to neurite elongation during NSC neuronal differentiation; by which to improve the prognosis of spinal cord injury in mice.

## 1. Introduction

SCI is a devastating injury of the CNS that often results in severe neurological dysfunction including paraplegia. A complex pathological process will occur in the injured spinal cord tissue once the injury occurs. For instance, once damage to the spinal cord occurs, homeostatic balance is altered drastically with a strong increase in host cell activation, and the release of an abundance of cytokines both at injury sites and in the circulation alters the neuron fate (Gregoire et al., 2015; Reis et al., 2017). Some research has indicated that the microenvironment that beneficial neurite outgrowth is essential for CNS remodeling, and neurological function rehabilitation during SCI (Gao et al., 2016; Wu et al., 2017; Nichols et al., 2018). Clinical practice has demonstrated that even small improvements in neurological function after spinal cord injury can provide large gains for patients. For this purpose, scholars have been searching for drugs and approaches that can effectively improve the recovery of neurological function after SCI.

HAP1 known as a protein identified could be interacted with protein huntingtin in Huntington’s disease (HD), and is mainly located in the hippocampus and caudate nucleus (Mackenzie et al., 2017). Two isoforms of HAP1, HAP1-A, and HAP1-B, have been identified in rodents based on their C-terminal sequences (Li et al., 1998; Mackenzie et al., 2017). Only one HAP1 has been detected in mammals, similar to HAP1-A in rodents. HAP1 has been demonstrated to be also associated with a variety of biological processes including intracellular transport, regulation of gene transcription, vesicular transport, endocytosis, signaling, and functional processes in many systems including the nervous system, digestive system, etc. (Li and Li, 2005; Li et al., 2019; Gong et al., 2020; Zhao et al., 2022). Evidence has shown that HAP1 can interact compactly with dynactin p150Glued, kinesin light chain, hepatocyte growth factor-regulated tyrosine kinase substrate, epidermal growth factor (EGF), and GABA, each of these elements is closely interrelated with the biological behavior of neurons (Woods and Seeley, 2006). And structurally interact closely with microtubules within the cellular context (Rong et al., 2007). Further, in HD-related studies, it has been demonstrated that HAP1 can counteract the inhibitory effect of mutant huntingtin on neurite outgrowth by stabilizing TrkA (McGuire et al., 2006; Rong et al., 2006). Based on its above-mentioned function in the CNS, it is speculated that hap1 possesses the potential to improve neurological function after spinal cord injury.

In this study, we tried to reveal the possible benefits of HAP1 administration in SCI. We found that HAP1 regulates tropomyosin receptor kinase A (TrkA)-mitogen-activated protein kinase (MAPK) phosphorylation to alter neurite elongation and ameliorate neurological function rehabilitation.

## 2. Results

### 2.1. HAP1 facilitates neurological function recovery after SCI and avail of Tuj *in vivo*

**Figure 1:**
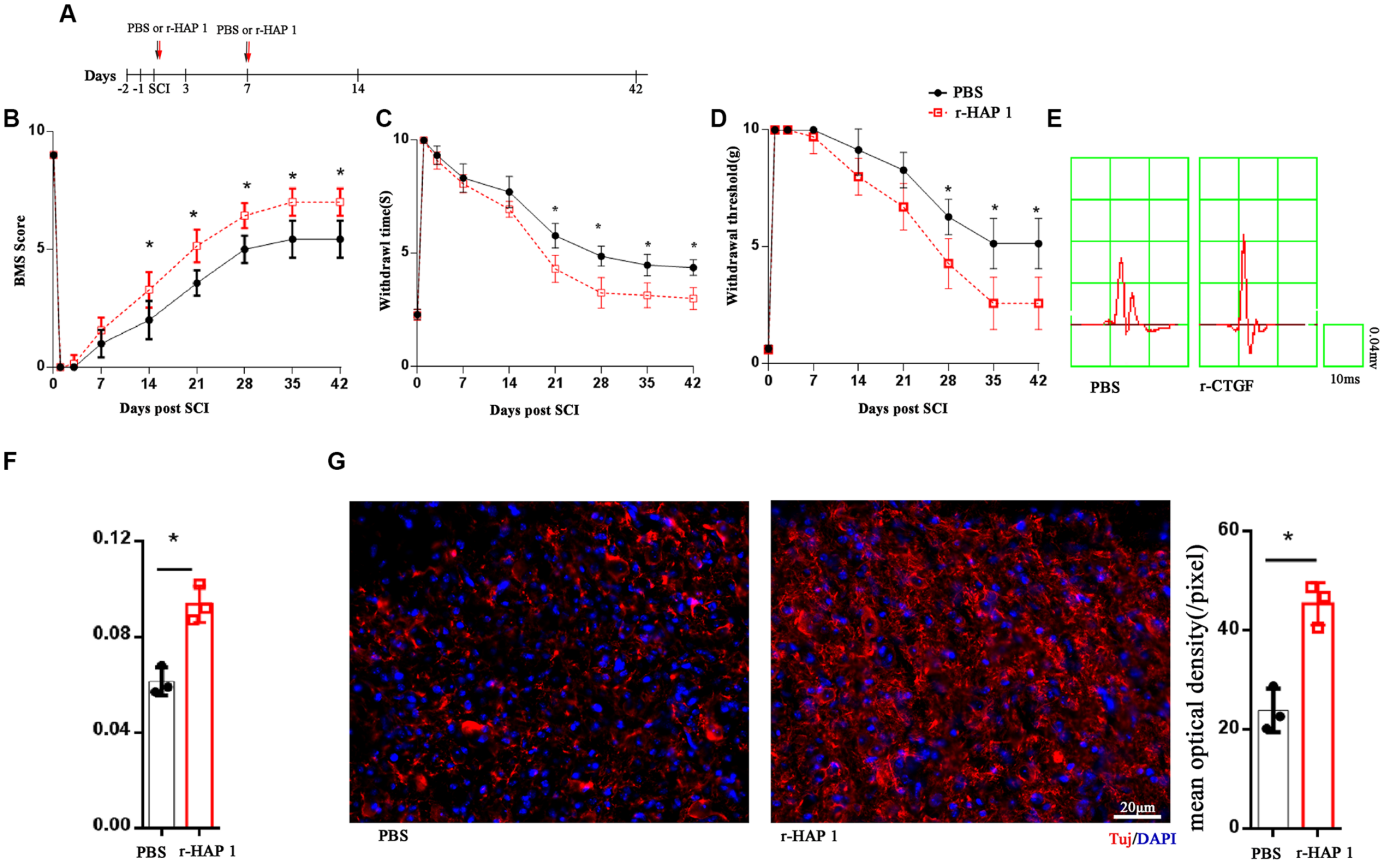
R-HAP1 facilitated neurological function recovery *in vivo*. **(A)** Schematic diagram of r-HAP1 and treatment protocol. **(B–F)** BMS score; temperature sensation threshold; tactile sensation threshold and cortical evoked motor potential of the SCI mice administered PBS and r-HAP1; *n* = 7 biological replicates/group. **(G)** Representative immunofluorescence result and quantification of Tuj in injured cord on the 14th day after SCI. Three independent biological replicates were performed. *Student’s *t* test *value of p* <0.05; r-HAP1, recombinant Huntingtin-associated protein 1; BMS, Basso Mouse Scale; SCI, spinal cord injury; IF, immunofluorescence; BrdU, 5-bromo-2′-deoxyuridine; DAPI, 4, 6-diamidine-2-phenylindole dihydrochloride. The white arrow: double positive cells.

To investigate the therapeutic effect of recombinant HAP1 (r-HAP1) in SCI, the SCI model was established with Allen’s weight drop apparatus. Then, r-HAP1 (2 mg/kg/day) was administered by intrathecal injection, respectively, on the day of SCI and on the seventh day after SCI (Figure 1A). The recovery of neurological function was analyzed by the restoration process of the Basso Mouse Scale (BMS) score, tenderness threshold, Warmth sensation threshold, and neurotic electrophysiology by cortical motor evoked potential (cEMP). The results showed that r-HAP1 can effectively improve the BMS score (Figure 1B), pain sensation (Figure 1C), warmth sensation (Figure 1D) and cEMP manifestation (Figures 1E,F) after SCI in mice, but has little effects on normal mice (Supplementary Figure S1).

To further investigate the pathological alterations of SCI-injured local spinal cord tissue after r-HAP1 administrations, by immunofluorescent the neuronal characteristic marker Tuj was identified, and we characterized the status of injured spinal cord surrounding neurons. The outcomes revealed that r-HAP1 significantly increased the expression of local Tuj in the area of injury (Figure 1G; Student test value of *p* < 0.05).

### 2.2. R-HAP1 promotes neurite elongation after neuronal differentiation of neural stem cells *in vitro*

**Figure 2:**
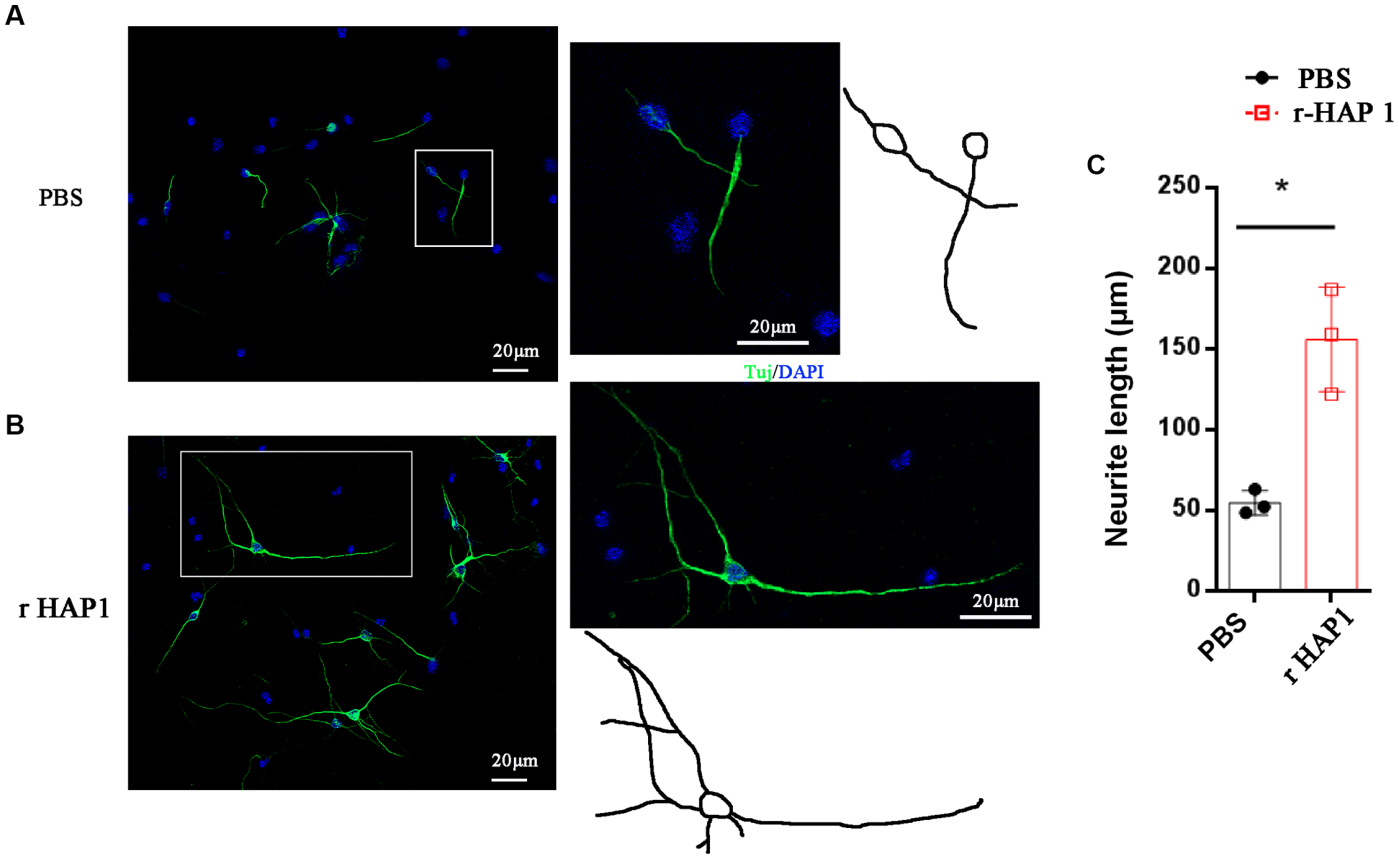
R-HAP1 promotes neurite outgrowth during NSC neuronal differentiation. **(A,B)** A typical result showing that r-HAP1 regulates neurite growth during neuronal differentiation with Tuj (green) staining for neurite. **(C)** Quantification of neurite length. *One-way ANOVA *value of p* < 0.05, three independent biological replicates were performed. r-HAP1, recombinant Huntingtin-associated protein 1; NSCs, neural stem cells.

To elucidate the effect of r-HAP1on growth of the axons, we isolated NSCs from spinal cord tissue from E14 mice. When NSCs were maintained with stemness in the proliferation phase, they grew as neurospheres and floated in the culture medium (Supplementary Figure 2A). As expected, these cells were positive for expression of the typical NSC marker nestin (Supplementary Figure 2B). R-HAP1 was added to the culture medium at a final concentration of 10 ng/mL, and the cells were incubated for 7–14 days for differentiation, the medium was changed every other day. The neurite length was calculated on the 7th day of neuronal differentiation. The results confirmed that r-HAP1 administration effectively promoted neurite outgrowth (Student test value of *p* < 0.05; Figure 2). Based on these findings, one may conclude that r-HAP1 is avail of the growth of neurite during neuronal differentiation in NSCs.

### 2.3. Signaling pathway analysis reveals TrkA-MAPK/ERK associated with the effects of r-HAP1 on NSCs

**Figure 3:**
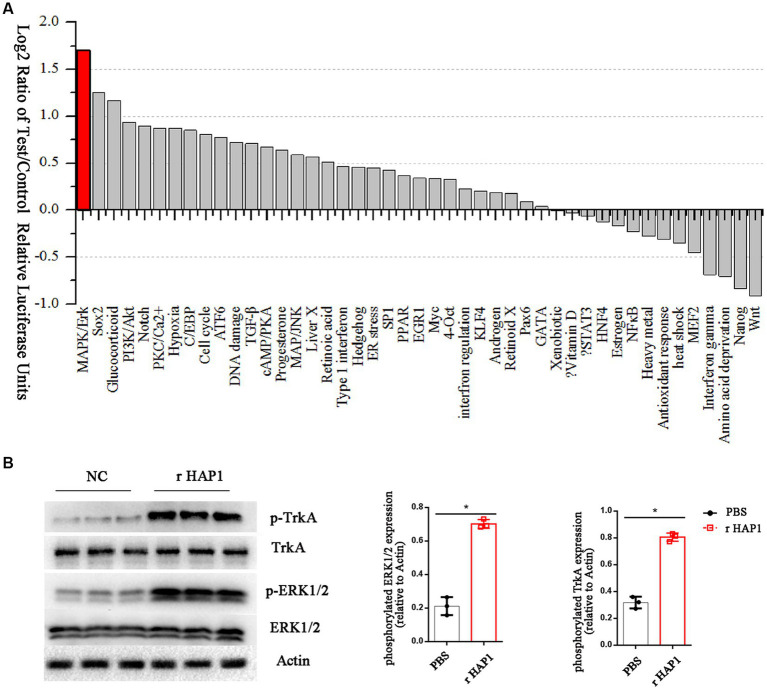
HAP1 changes the ERK signaling pathway in NSCs by TrkA. **(A)** The signal pathway array results of HAP1-regulated signaling manifestation in NSCs, Listed by log2-fold change in descending order. **(B)** Representative western blot result and semi-quantification of TrkA and ERK phosphorylation. *Student’s *t* test *value of p* < 0.05. r-HAP1, recombinant Huntingtin-associated protein 1; NSCs, neural stem cells; ERK, extracellular signal-regulated kinase.

To gain more information on the effects of HAP1 in neurite growth, we further investigated the signaling mechanisms involved in HAP1-mediated regulation of NSCs. We screened the levels of signaling pathway changes in r-HAP1-treated NSCs with a Cignal Finder Reporter Array. The results showed that certain pathways were significantly activated, including the mitogen-activated protein kinase/extracellular regulated protein kinase (MAPK/ERK) pathway, the Sox2 pathway the Glucocorticoid pathway (log2 > 1). Most of the signaling pathway changes were not significant (Figure 3A).

The MAPK/ERK pathway was further probed by Western blotting as it has been reported that HAP1 activates the tyrosine kinase receptor TrkA and stimulates its phosphorylation (Rong et al., 2006; Su et al., 2018). To verify the hypothesis that HAP1 promotes TrkA phosphorylation by activating the MAPK/ERK pathway, we examined the phosphorylation of TrkA and ERK1/2 in our experimental conditions. The western blotting results showed a significant increase in the phosphorylation levels of TrkA and ERK1/2 in r-HAP1-treated NSCs than the PBS group (Student test value of *p* < 0.05, Figure 3B).

**Figure 4:**
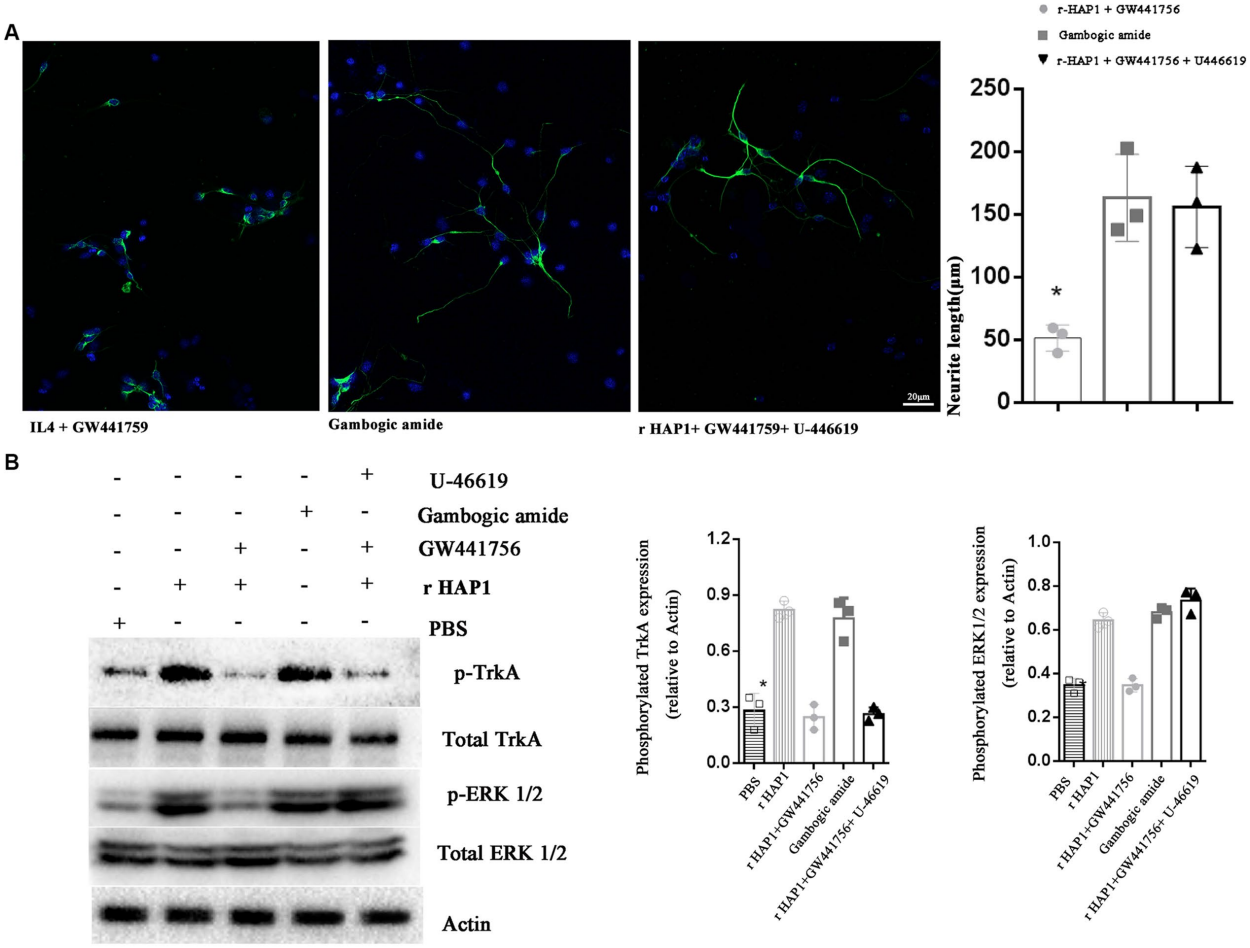
Blocking TrkA activation inhibits the effect of HAP1 on NSCs. **(A)** Representative results of neurite growth and Neurite length quantification during neuronal differentiation in NSCs exposed to r-HAP1 and GW441756; gambogic amide; r-HAP1, GW441756 and U-46619. **(B)** Typical Western blot and semi-quantification results from three independent experiments showing the phosphorylation of TrkA and ERK. *One-way ANOVA *value of p* < 0.05, three independent biological replicates were performed.

To further define the molecular mechanism by which HAP1 promotes neurite elongation after neuronal differentiation of neural stem cells, TrkA selective inhibitor GW441756 (2 μM; Selleck, China) (Jung et al., 2013), TrkA selective activator gambogic amide (0.5 μM, Santa Cruz Animal Health, USA) (Jang et al., 2007) and ERK activator U-46619 (10 nM; Santa Cruz, USA) (Nishihara et al., 2000) were used. The lengths of neurites after 7 days of neuronal differentiation were calculated. In the r-HAP1 + GW441756 group, the neurite length was significantly lower than gambogic amide group and r-HAP1 + GW441756 + U-46619 group (Figure 4A; one-way ANOVA with Turkey’s multiple comparisons test value of *p* < 0.05); Results indicated that the effect of HAP1 on neurite elongate can be inhibited by GW441756, and reversed by ERK1/2 activation. At the same time, gambogic amide could mimic the function of HAP1 in regulating neurite elongate after neuronal differentiation of neural stem cells.

Further, the Western blot results were in accordance with the results for neurite elongation. GW441756 significantly blocked TrkA and ERK1/2 phosphorylation (Figure 4B; one-way ANOVA with Tukey’s multiple comparison test value of *p* < 0.05 versus r-HAP1 group). From previous results, we concluded that gambogic amide could simulate the function of r-HAP1. Western blot results further illustrated that gambogic amide significantly overregulated the phosphorylation of TrkA and ERK1/2 (Figure 4B; one-way ANOVA with Tukey’s multiple comparisons test value of *p* < 0.05 versus PBS group). U-46619 caused ERK phosphorylation when TrkA was inactivated. These data strongly support and confirm our hypothesis that the HAP1-TrkA-MAPK/ERK signaling pathway regulates the neuronal differentiation of NSCs and promotes neurite outgrowth during neuronal differentiation.

### 2.4. Trka-MAPK/ERK is essential for r-HAP1 to promote axonal neurite elongation

**Figure 5:**
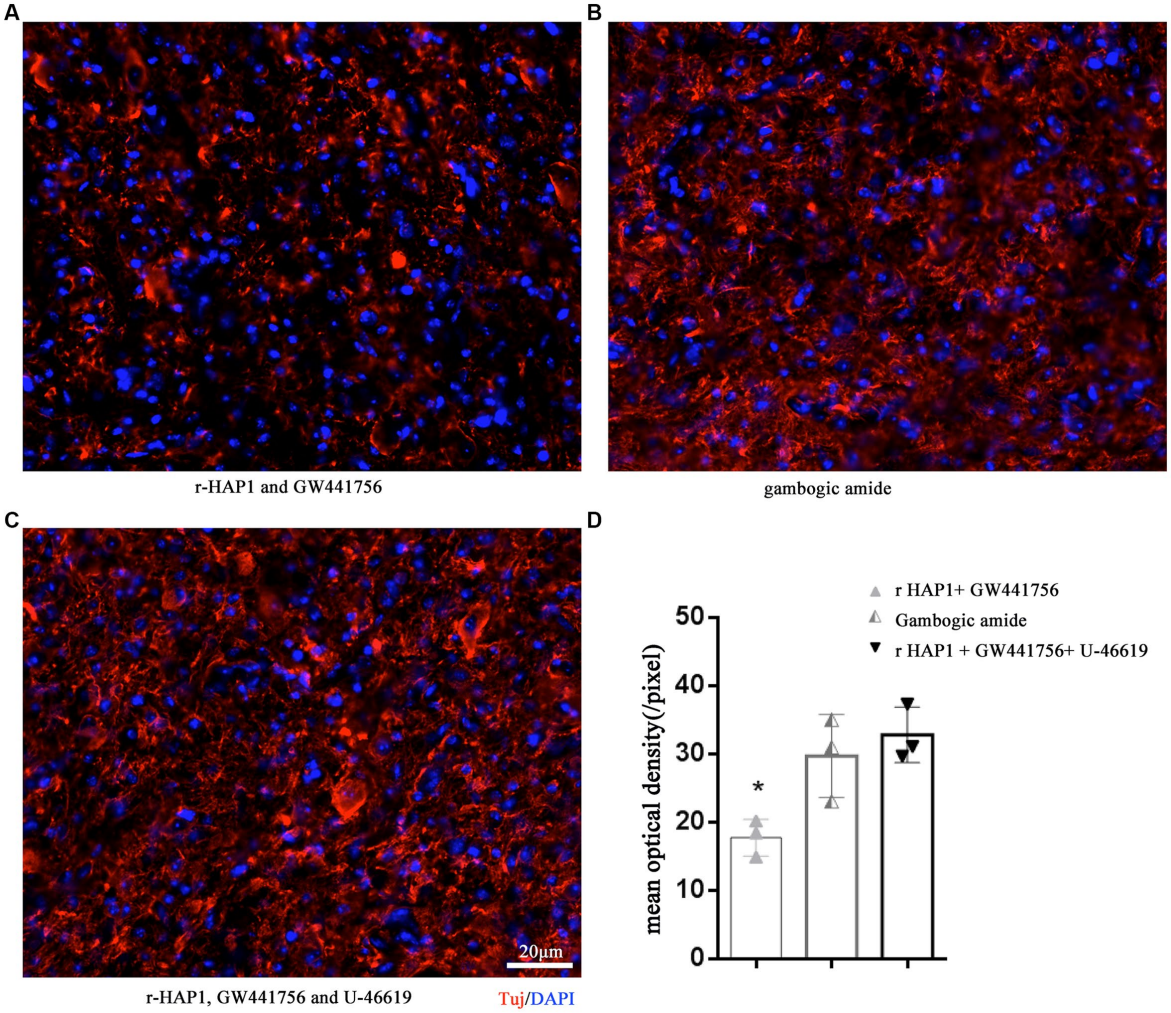
TrkA-MAPK/ERK is essential for r-HAP1 promote axonal neurite elongation. Representative results of Tuj regeneration after SCI with the administration of r-HAP1 and GW441756 **(A)**; gambogic amide **(B)**; r-HAP1, GW441756 and U-46619 **(C)**, and the semi-quantification results **(D)**. r-HAP1, recombinant Huntingtin-associated protein 1; ERK, extracellular signal-regulated kinase.

To further validate the role of the HAP1-TrkA-MAPK/ERK signaling pathway in axonal neurite elongation *in vivo*, GW441756, gambogic amide, and U-46619 were grouped according to *in vitro* tests and injected in intralumbar. Tuj was identified on the 14th day after SCI modeling. The result was similar to the *in vitro* outcomes, in the r-HAP1 + GW441756 group the Tuj was significantly lower than gambogic amide group and the r-HAP1 + GW441756 + U-46619 group (Figure 5; one-way ANOVA with Tukey’s multiple comparisons test value of *p* < 0.05).

### 2.5. Trka-MAPK/ERK is involved in neurological rehabilitation after SCI

**Figure 6:**
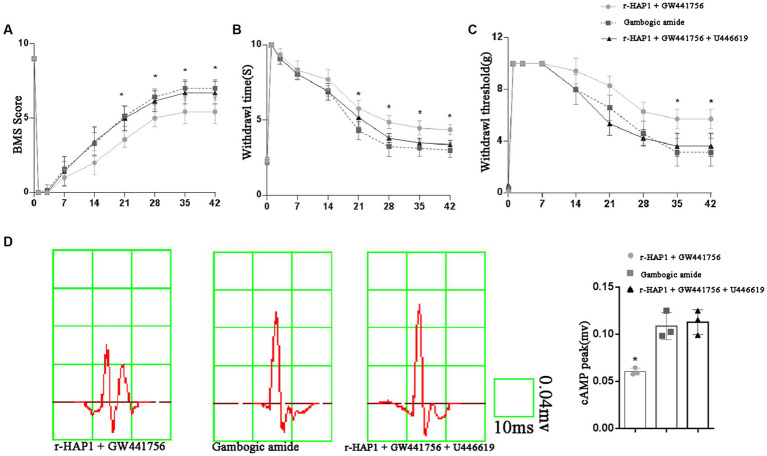
TrkA-MAPK/ERK involved in neurological rehabilitation after SCI. BMS score **(A)**. Temperature sensation threshold **(B)**. Tactile sensation threshold **(C)** and cortical evoked motor potential **(D)** of the SCI mice administered with r-HAP1 and GW441756; gambogic amide; r-HAP1, GW441756, and U-46619. *One-way ANOVA *value of p* < 0.05, three independent biological replicates were performed.

To verify the role of TrkA-MAPK/ERK in neurological function, The BMS score, pain sensation, warmth sensation, and cEMP manifestation were examined again with the administration of GW441756, gambogic amide, and U-46619. The BMS score result shows that r-HAP1 administrated with the GW441756 group was significantly lower than the gambogic amide group and r-HAP1 + GW441756 + U-46619 group (Figure 6A). Pain sensation, warmth sensation, and cEMP manifestation (Figures 6B–D) are consistent with BMS score results. Meanwhile, the administration of GW441756, gambogic amide, and U-46619 had no impact on the neurological function of healthy mice (Supplementary Figure S2). Combining the above-described results, it can be concluded that TrkA selective inhibitor GW441756 will block the effect of r-HAP1 to promote neurological rehabilitation, while TrkA selective activator gambogic amide can mimic the therapeutic effect of r-HAP1 on SCI mice; ERK activator U-46619 can restore the blocking effect of TrkA selective inhibitor GW441756 on r-HAP1.

## 3. Discussion and conclusion

In this study, we demonstrated the therapy effects and potential mechanism of HAP1 acts on spinal cord injury in mice. With the administration of HAP1, the neurological function of the SCI mice was significantly improved. HAP1 is a protein first identified as an htt-binding partner, and it is abundantly expressed in the brain. As reported, HAP1 acts as a scaffold protein combine to the expanded polyglutamine repeat in HD (Zhao et al., 2022). In the thalamus, HAP1 has been shown to be closely associated with feeding behavior by interacting with the feeding-inhibitory actions of insulin in the brain (Woods and Seeley, 2006). The loss of HAP1 will lead to decreased feeding actions and result in malnutrition, dehydration, and even death (Woods and Seeley, 2006; Liu et al., 2020). Further, HAP1 loss was shown to lead to the degeneration of neurons within the striatum, which gives a potential for HAP1 a cure for CNS disease (Liu et al., 2020). On the whole, the current functional studies of HAP1 are mainly focused on HD and related neurodegenerative disorders, with little understanding of other dimensions. Here, we concentrated on the role of HAP1 in SCI.

Injuries to SCI are usually segregated into primary and secondary damage (Tran et al., 2018). Primary injury can cause the breakage of neural axons directly; while secondary injury caused by inflammation, ischemia–reperfusion, and excitatory amino acids toxicity will further lead to the reduction of neurons and neural protrusions (Anjum et al., 2020). Although the rehabilitation of neurological function after SCI is related to various factors such as the degree of injury and the local pathological changes after the injury, neural axons serve as the structural basis for transmitting the neural signals in the CNS and are closely related to the state of neural function after SCI (Tran et al., 2018; de Freria et al., 2021). With the administration of r-HAP1 in cultured primary NSCs and SCI mice, we have confirmed synchronously both *in vitro* and *in vivo* that r-HAP1 will substantially benefit the neurite growth on NSCs during neuronal differentiation and axonal regeneration at the injured site of the spinal cord.

As HAP1-related studies are still insufficient, its related signaling pathway is less reported. We delved deeper into the role of HAP1 in SCI treatment and further confirmed the changes in the signaling pathways of NSCs after HAP1 manipulation with a pathway array. First, the MAPK-ERK1/2 signaling pathway was markedly upregulated. The glucocorticoid, Sox2, PI3K/Akt, and Notch pathway also upregulated significantly. Secondly, pathways including Wnt, Nanog are downregulated.

The function of Hap1 associated with the glucocorticoid signaling pathway is to participate in the hypothalamic response to abnormal stress-related disorders including degenerative diseases, such as HD and Parkinson’s, where abnormally high chronic cortisol levels were reported (Karadima et al., 2012; Chen et al., 2023). Disruption of the regulatory homeostasis of glucocorticoid by Hap1 is considered a major risk factor for neurodegenerative diseases and neuropsychiatric disorders (Chen et al., 2020). Furthermore, regulation associated with the Notch signaling pathway is focused primarily on neurogenesis (Sheng et al., 2008; Xiang et al., 2014). In Drosophila, it was demonstrated that using the targeted expression of Hap1 throughout the dorsal notum, full-length Hap1 reduced the density of dorsal notum macrochaetae, and the lack of a functional lipid-binding ANTH structural domain led to an increase in microchaetaer density (Sharma et al., 2017). Drosophila notum develops from neural precursor tissue. Studies of embryonic stem cell knockdown of Hap1 suggest involvement in early neurogenesis via Notch, Hes1, and Hes5-related mechanisms and can affect early neurogenesis and oligodendrocyte precursor cell development (Dolan et al., 2019; Chen et al., 2022). The association and Function related to Sox2, PI3K/Akt, Wntg, and Nanog signaling pathways lack appropriate reports. For our study, we have demonstrated that HAP1 will promote MAPK/ERK phosphorylation through the TrkA signaling pathway. Consistent with the HD-related study, HAP1 exhibited a stabilizing effect on neural axons via TrkA. Furthermore, it has been reported that MAPK-ERK1/2 pathway activation in NSCs is closely related to the process of neurite outgrowth (Wu et al., 2016; Yang et al., 2021), and our findings also support this point.

TrkA is a dual-receptor system that is known to transduce neurotrophin signals and is one of the high-affinity receptors of nerve growth factor (NGF) (Tomlinson et al., 2017). NGF activates TrkA to promote the differentiation of NSCs and stimulates neurite outgrowth (Turney et al., 2016). The experimental results provide evidence that HAP1 activates TrkA phosphorylation in NSCs. By assessing the binding and activation of TrkA, we further determined that TrkA plays an important role in the process of HAP1-guided neurite growth. Inhibitors of TrkA could effectively block the HAP1-mediated promotion of neurite growth and neurological function recovery, and the TrkA activator could mimic the function of HAP1. A previous study has revealed that HAP1 is colocalized with TrkA (Rong et al., 2006). Given the findings listed above, we conclude that HAP1 can directly bind to TrkA in NSCs and activate the MAPK/ERK signaling pathway.

Notably, one shortcoming of our study is that we only inspected the regeneration of Tuj after HAP1 treatment but did not pursue further its effect on the ultrastructure of axons, terminal vesicles, synapses, etc. An in-depth investigation of the changes in these structures will provide more clarification of the therapeutic mechanism of HAP1 on SCI. Second, this research did not accomplish the observation that HAP1 is on the differentiation of NSCs. This will also be the major focus and direction of our subsequent research.

In summary, we provide evidence that HAP1 ameliorates neurological function in SCI mice, by facilitating neural axon regeneration *in vivo* and promoting neurite elongation in the neuron differentiation of NSCs *in vitro*. TrkA plays an important role in this process by activating MAPK-ERK1/2 signaling.

## 4. Experimental procedure

### 4.1. SCI model in mice

C57BL/c mice aged approximately 6–8 weeks were provided by the Department of Laboratory Animals of Central South University. All animal breeding, care, and testing procedures were approved by the Animal Care and Use Ethics Committee of Central South University Xiangya Hospital. All mice were anesthetized with ketamine (60 mg/kg) and xylazine (10 mg/kg) by intraperitoneal injection. With skin preparation, disinfection, and positioning, the skin, subcutaneous muscle, and lamina were cut at the T10 spinous process to reveal the spinal cord. A model of incomplete spinal cord contusion was established using a modified Allen’s method with an impact force of 10 g × 25 mm. The wound was rinsed, the incision was closed layer by layer, and the wound was covered. R-HAP1 (2 μg r-HAP1 diluted in 0.5 μL PBS) was administrated by intralumbar delivery. All mice were housed in an animal laboratory at an ambient temperature of 22 to 24°C and a relative humidity of 60 to 80%. After the operation, the mice were kept in separate cages, with standard food and drinking water offered *ad libitum*. An intramuscular injection of penicillin 2 WU/Bid was routinely given to prevent infection 3 days after surgery. The bladder was squeezed 2 to 4 times daily to aid urination until the micturition reflex was restored.

### 4.2. Intralumbar injection

Intralumbar injection was referred to Bey et al. (2017). Briefly as follows, a 30-gauge beveled needle was connected to a 10-μl syringe (Hamilton, Ghiroda, Romania), and the needle was inserted between lumbar vertebra 4 and lumbar vertebra 5; when the needle was properly inserted there will be a tail flick reflex, r-HAP1, gambogic amide, and PBS were epidurally administered via a syringe pump injection.

### 4.3. Behavioral tests

The behavioral tests were performed as previously described. Briefly, the locomotive function was evaluated by Basso Mouse Scale (BMS). The von Frey filament test was used to test the sensation threshold of pain, and the unilateral Hargreaves thermal test was used to test the sensation threshold of warmth. The cortical evoked motor potential examination was used to identify the Neuro-conduction capacity. All of this was tested by two independent assessors who were blinded to the experimental design and who were familiar with those assessment methods.

### 4.4. NSC isolation, culture, and differentiation

NSCs were isolated from the spinal cords of fetal mice on embryonic day 14.5 (E14.5). The spinal cords harvested from 3–4 E14.5 embryos were cut up, dissociated with 1 mL micropipettes, digested with the same volume of Accutase (StemPro, US) for 10 min, and centrifuged at 1,000 rpm for 5 min. The pellets were resuspended in a mouse NSC culture medium (Cyagen, China). The cells were seeded into T-25 culture flasks and incubated in a cell incubator at 37°C in a humidified atmosphere with 5% CO2. Single cells grew and accumulated into neurospheres for 5 days. For passaging, neurospheres were collected by centrifugation and then digested with accutase for 1–2 min at 37°C. The neurospheres were aspirated with 1 mL micropipettes approximately 20 times. The NSCs were reseeded with NSC culture medium at a density of 20 × 10^4^ cells/ml into T-25 culture flasks.

For differentiation, NSCs were seeded onto poly-lysine-coated (Sigma, USA) slides in 24-well cell culture plates and grown with different differentiation media for 7 to 14 days. For neuronal differentiation, NSCs were cultured in neuronal differentiation medium composed of neurobasal medium (Gibco, US), 2% B-27 (Gibco, US), 2 mM L-glutamine (Gibco, US), 2 μM all-trans retinoic acid (Sigma, US), 5 μM forskolin (MedchemExpress, China) and 0.05 g/L penicillin/streptomycin (Gibco, US). To identify the neurite outgrowth efficiency, NSCs were seeded at a density of 2.5 × 10^4^ cells/well and to verify the efficiency of differentiation, NSCs were seeded at a density of 10 × 10^4^ cells/well. To differentiate into astrocytes, NSCs were cultured in Dulbecco’s modified Eagle’s medium (DMEM)-F12/GlutaMAX (Gibco; US), 2% B-27, and 10% fetal bovine serum (Gibco, US). For oligodendrocyte differentiation, NSCs were cultured in an oligodendrocyte differentiation medium containing DMEM-F12/GlutaMAX, 2% B-27, and 200 ng/mL insulin-like growth factor 1 (Cyagen, China).

### 4.5. Immunofluorescence analysis

Cells were seeded onto cell culture plates; frozen sections were fixed with 4% paraformaldehyde for 10 min at room temperature, washed with phosphate-buffered saline (PBS) three times, and permeabilized with 0.1% Triton X-100 in PBS for 15 min. For BrdU (5-bromo-2′-deoxyuridine) labeling, the cells and sections were treated with 2 M HCl for 30 min and neutralized with 0.1 M barium tetraborate for 20 min. The cells and sections were blocked with 4% BSA in PBST for 1 h and then incubated with the appropriate primary antibody against the following: nestin (ab6142, Abcam, 1:200 dilution), Tuj (ab18207, Abcam, 1:400 dilution), O4 (MAB345, Millipore, 10 μg/mL), GFAP (ab7260, Abcam, 1:1,000 dilution), NeuN (ab104225, Abcam, 1:1,000 dilution), and BrdU (ab1893, Abcam, 1:400 dilution) at 4°C overnight. The cells were washed three times with PBS, incubated with the appropriate secondary antibody (Alexa Fluor^®^ 647 Goat Anti-Rat IgG, ab150167, Abcam, 1:500 dilution; Alexa Fluor^®^ 488 Goat Anti-Rabbit IgG, ab150077, Abcam, 1:500 dilution; Alexa Fluor^®^ 647 Goat Anti-Rabbit IgG, ab150083, Abcam, 1:500 dilution; Alexa Fluor^®^ 488 Rabbit Anti-Sheep IgG, ab1501817, Abcam, 1:500 dilution; Alexa Fluor^®^ 488 Goat Anti-Mouse IgG H&L, ab150113 Abcam, 1:500 dilution) at room temperature for 90 min, and stained with DAPI.

To calculate the length of the neurite (Wang et al., 2017), representative regions were harvested by Zeiss microscope. The neurite was manually traced by ImageJ software, and a schematic diagram of neurons and neurites was portrayed. The total neurite length of each neuron was measured by ImageJ software, and the average length of a random zone from each biological replicate was calculated. The target regions of the cell slide or the representative sections were randomly captured, and the positive cells were counted independently by two people. Each experiment was biologically repeated three times.

### 4.6. Pathway analysis

To investigate signal changes, a Cignal Finder Reporter Array plate (Qiagen, US) was used according to the manufacturer’s instructions. Briefly, 50 μL of NSC complete medium was added to each well of the Cignal Finder Array plate. The side of the plate was gently tapped to resuspend the constructs. Then, the plate was slightly rocked back and forth and left and right before being incubated for 5 min at room temperature. Next, 0.6 μL of attractions in 50 μL of NSC complete medium was added to each individual transfection. The plate was incubated for 20 min at room temperature to allow complex formation to occur. Simultaneously, NSCs were isolated as described previously. The cells were suspended in 5,400 μL of complete medium at a density of 20 × 10^4^ cells/ml. A total of 50 μL of cell suspension was added to each well containing construct-attractive complexes. The plate was mixed gently by rocking. The cells were incubated at 37°C in a 5% CO2 incubator for 12 h, and then recombinant HAP1 (r-HAP1, 15 ng/mL; BIOMATIK) was added. Twelve hours later, a luciferase assay was performed using a dual-luciferase reporter assay system. The luminescence signal was quantified by following the manufacturer’s instructions by normalizing the ratios of cellular responses to r-HAP1 (firefly luminescence signal) to the nonspecific responses to PBS (renilla luminescence signal). The r-HAP1-treated cells were compared with the PBS-treated control cells, and the results were plotted as a log2-fold change. If log2 > 1, the difference was considered significant.

### 4.7. Western blot analysis

After cells were harvested, ice-cold Dulbecco’s PBS (DPBS) was used to wash cells three times, and RIPA buffer with protease inhibitor and phosphatase inhibitor was used for cell lysis. Cellular protein was collected by centrifugation of the cell lysates at 12,000 rpm for 10 min. The protein concentration was measured with a BCA protein quantitation kit (Thermo Scientific, USA). Total proteins were separated by 10% SDS-PAGE and then transferred to nitrocellulose membranes. The membranes were blocked with 5% nonfat dry milk in tris-buffered saline/0.5% Tween. Then, the cells were incubated with the appropriate primary antibody against the following: ERK 1/2 (ab184699, Abcam, 1:10,000 dilution), phosphorylated ERK1/2 (ab76299, Abcam, 1:10,000 dilution), TrkA (ab76291, Abcam, 1:5,000 dilution), and phosphorylated TrkA (ab193229, Abcam, 1:1,000 dilution) before being incubated with the appropriate secondary antibody (Goat Anti-Rabbit IgG, ab216773, Abcam). Pierce™ ECL plus western blotting substrate was used to detect the proteins.

### 4.8. Statistics

All data are expressed as the mean ± standard deviation (SD) of three independent experiments. The results were analyzed by a two-tailed Student’s test for comparisons between two groups, and one-way ANOVA with Tukey’s multiple comparisons test for multiple groups. Statistical and graphical analyses were performed using the software GraphPad Prism 6 (GraphPad Software, USA). *p* < 0.05 indicated statistical significance.

## Data availability statement

The original contributions presented in the study are included in the article/Supplementary material, further inquiries can be directed to the corresponding author.

## Ethics statement

The animal study was reviewed and approved by Ethics Committee of Hunan Children’s Hospital.

## Author contributions

LT was focused on the design of the work the study, mainly working on an agreement to be accountable for all aspects of the work in ensuring that questions related to the accuracy appropriately investigated and resolved, and final approval of the version to be published. LM contributed to the acquisition, analysis, interpretation of data for the work, drafting the work, and revising it critically. SQ mainly contributes to drafting the work and revising it critically. All authors contributed to the article and approved the submitted version.

## Funding

This study was supported by Hunan Provincial Natural Science Foundation of China (No. 2023JJ40345; No. 2023JJ40006). Scientific Research Project of Hunan Provincial Health Commission (No. 2306028547).

## Conflict of interest

The authors declare that the research was conducted in the absence of any commercial or financial relationships that could be construed as a potential conflict of interest.

## Publisher’s note

All claims expressed in this article are solely those of the authors and do not necessarily represent those of their affiliated organizations, or those of the publisher, the editors and the reviewers. Any product that may be evaluated in this article, or claim that may be made by its manufacturer, is not guaranteed or endorsed by the publisher.
