# Supplementary material for: Huntingtin-associated protein 1 ameliorates neurological function rehabilitation by facilitating neurite elongation through TrKA-MAPK pathway in mice spinal cord injury
Source: Front Mol Neurosci. 2023 Aug 7;16:1214150. doi: 10.3389/fnmol.2023.1214150 (PMC10442162; doi:10.3389/fnmol.2023.1214150)
Supplement: Supplementary file 1 [file Image_1.pdf]

Supplementary figure 1

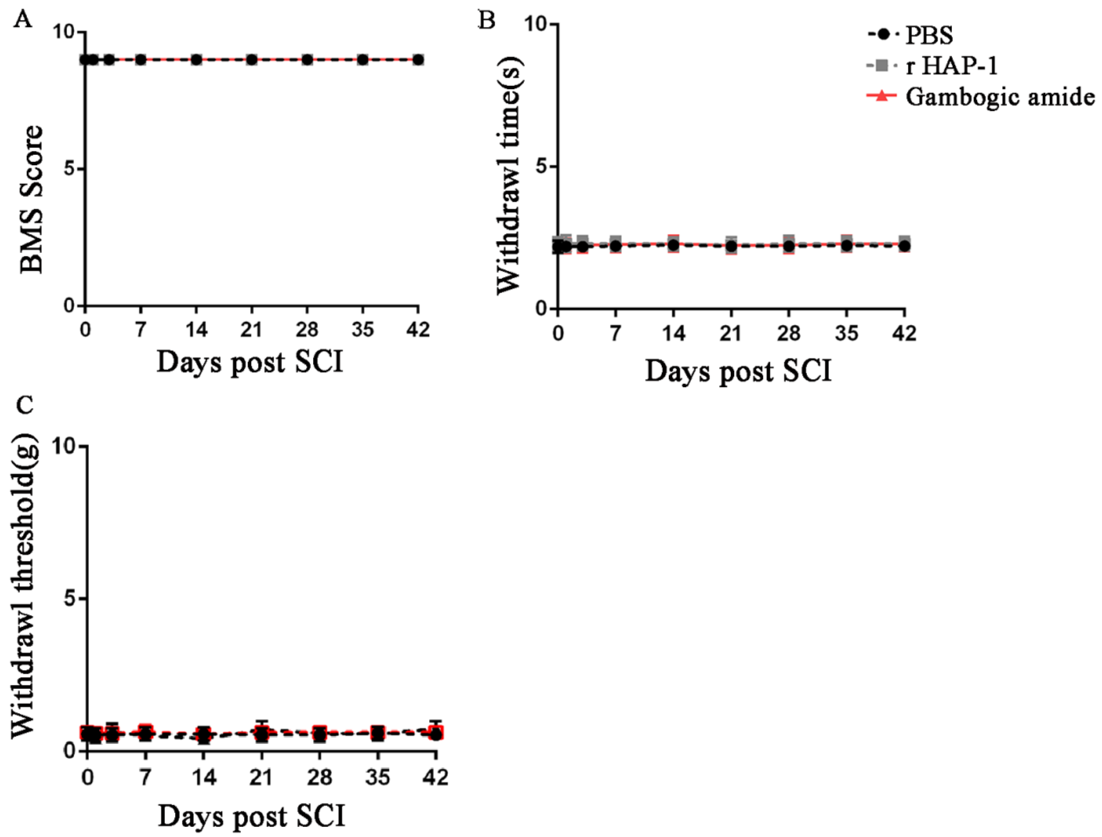

Intrathecal injection of r-HAP1 and Gambogic amide did not affect the hinder limb neurological function on normal mice. A, BMS score; B, temperature sensation threshold; C, tactile sensation threshold.
