# Supplementary material for: Huntingtin-associated protein 1 ameliorates neurological function rehabilitation by facilitating neurite elongation through TrKA-MAPK pathway in mice spinal cord injury
Source: Front Mol Neurosci. 2023 Aug 7;16:1214150. doi: 10.3389/fnmol.2023.1214150 (PMC10442162; doi:10.3389/fnmol.2023.1214150)
Supplement: Supplementary file 2 [file Image_2.pdf]

\

Supplementary figure 2

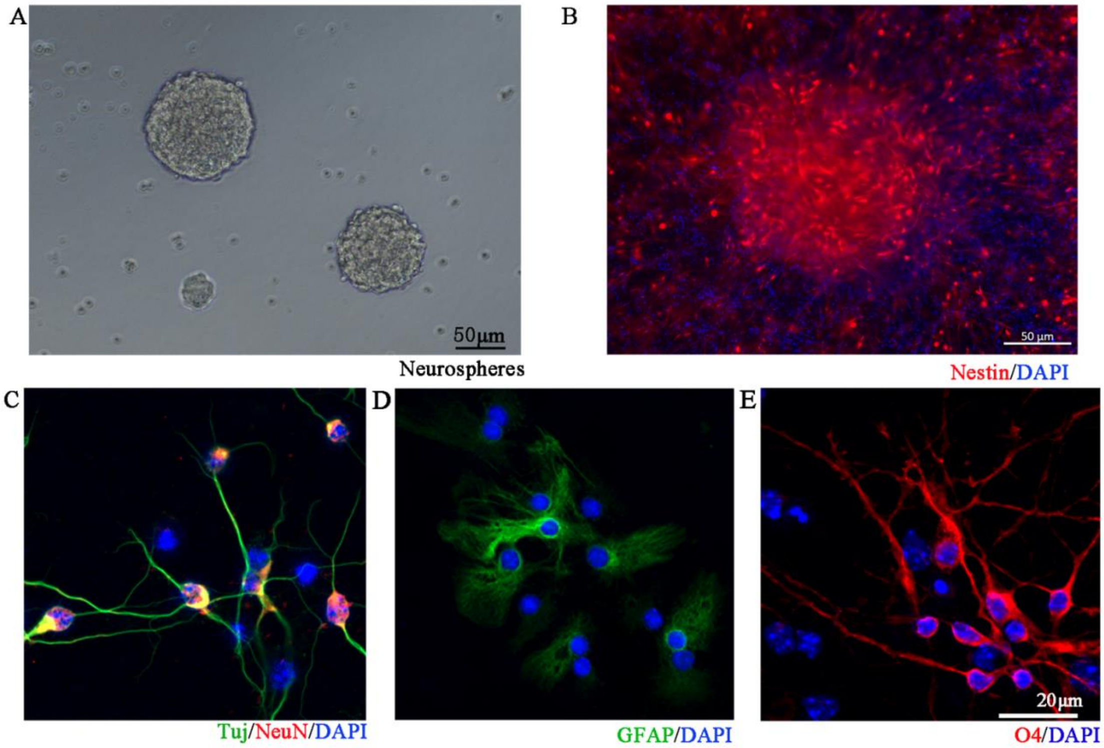

A, Suspension-cultured neurospheres. B, Nestin (red) labeling of adherent cultured neurospheres in poly-lysine-coated slides. C, Neuron differentiation; D, astrocyte differentiation and E, oligodendrocyte differentiation of NSCs.
